# Ammonia release from hydrolysed animal and food waste by the hop endophyte, *Pantoea agglomerans*

**DOI:** 10.1007/s11274-025-04720-0

**Published:** 2025-12-02

**Authors:** Tomas Hasek, Frantisek Kastanek, Tatiana Anatolievna Smirnova, Olga Solcova, Stepanka Kuckova, Barbora Branska, Karel Melzoch, Petra Patakova

**Affiliations:** 1 Department of Biotechnology, University of Chemistry and Technology Prague, Technicka 5, Prague 6, 166 28 Czechia; 2 Research Group of Catalysis and Reaction Engineering, Institute of Chemical Process Fundamentals of the Czech Academy of Sciences, Rozvojova 135, Prague 6, 165 00 Czechia; 3 Department of Biochemistry and Microbiology, University of Chemistry and Technology Prague, Technicka 3, Prague 6, 166 28 Czechia

**Keywords:** Ammonification, *Pantoea agglomerans*, Plant biostimulant, Animal waste, Chicken feather, Spent brewer’s yeast, Hydrolysis

## Abstract

The food industry and agriculture produce large amounts of waste that can be used in many upcycling applications. An interesting solution may be to return the necessary nutrients for plants to the soil and improve plant growth at the same time by using waste from animal production and food processing as a substrate for endophytic bacteria. Endophytic bacteria that naturally colonize plants can help plants absorb nutrients and may produce substances that promote plant growth. Animal waste (feathers, chicken or fish scraps) and spent brewer’s yeast rich in protein were hydrolysed into a mixture of soluble peptides and amino acids. The hydrolysates were used for the release of ammonium ions by the bacterium *Pantoea agglomerans* DBM 3797, isolated from hops. The release of ammonium ions is a result of excess nitrogen over carbon during the growth of bacteria that use amino acids as a source of carbon and energy. Ammonium ions are a form of nitrogen that is very easily utilized by plants. High concentrations of ammonium ions in g/L were achieved with both acidic and alkaline chicken feather hydrolysates and in spent brewer’s yeast autolysate, with the highest ammonium ions per unit of original material (41 mg/g) being released from the feather alkaline hydrolysate. It was also confirmed that differences in waste types and their processing have a significant effect on ammonia release, which is important for the development of a new biofertilizer. Proof of concept test on 3-week-old lettuce seedlings showed that addition to the soil of hydrolysate containing *P. agglomerans* significantly promoted the growth of the plant root system.

**Graphical abstract:**

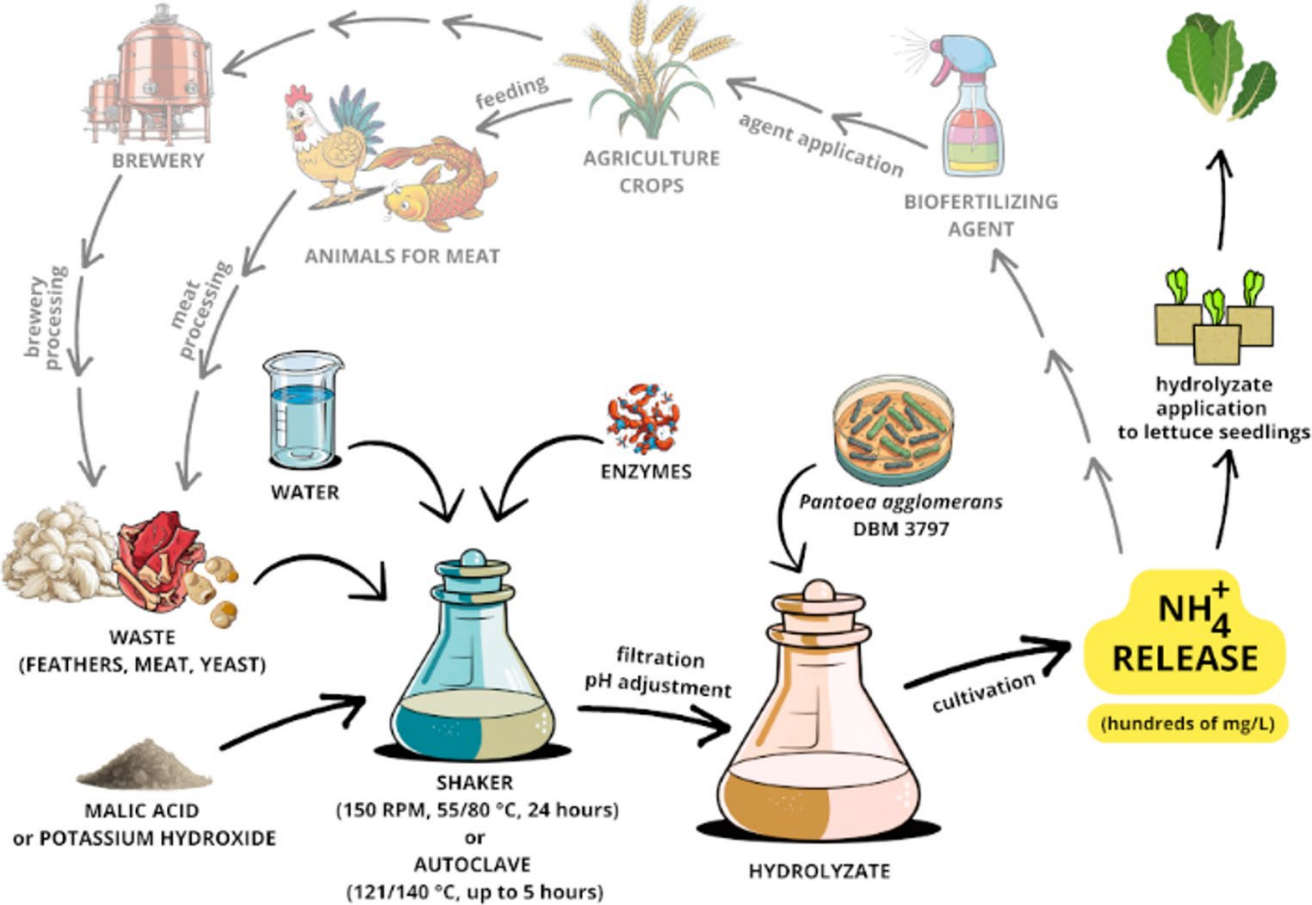

**Supplementary Information:**

The online version contains supplementary material available at 10.1007/s11274-025-04720-0.

## Introduction

In the current era of climate change, which is also associated with unexpected periods of drought, there is a need to develop new products (biostimulants) that will help plants survive these periods and ensure sufficient harvests (Girotto & Cossu 2017). Biostimulants can be produced from animal waste, the amount of which is constantly increasing. The total amount of chicken feathers from chicken farming was estimated in 2021 (Mishra et al. 2023) to be 102 million tons worldwide. The basic method for obtaining biostimulants from animal and food waste rich in proteins is the hydrolysis of insoluble proteins into a mixture of soluble peptides and amino acids. The effect of protein waste hydrolysates on plant growth includes a beneficial effect on primary and secondary plant metabolism, which makes plants less susceptible to abiotic stress, including drought (Colla et al., 2015). The peptides in these hydrolysates can exhibit various biological activities, beyond simple plant growth promotion, such as antimicrobial, insecticidal, or herbicidal effects (Zhang et al. 2023).

Not only biostimulants but also fertilizers play a significant role in agriculture and represent a powerful tool for continuous productivity throughout the seasons. Ammonification of organic compounds, i.e., release of ammonia from compounds such as amino acids, is an important part of the natural nitrogen cycle (Bernhard 2010) which mediates nitrogen uptake to plants and microorganisms by converting organic nitrogen into inorganic one. This process can lead to the replacement of inorganic nitrogen fertilizers based on ammonium salts with organic waste from animal production and food processing, applied together with ammonifying bacteria. Plants absorb nitrogen in the form of nitrate and ammonium ions, with the ideal ratio of these forms of inorganic nitrogen depending on the plant, its stage of development, soil, climate, and other factors (Chen et al. 2024).

Ammonification occurs during laboratory cultivation of bacteria in LB (lysogenic broth) medium. LB culture medium is based on the use of amino acids rather than carbohydrates as carbon sources, with the release of excess nitrogen in the form of ammonium ions into the culture medium (Sezonov et al. 2007). However, LB medium uses relatively expensive complex components (tryptone and yeast extract), which might be replaced by various hydrolysates of animal waste and spent brewer’s yeast. Replacing expensive complex components of microbial cultivation media with cheaper alternatives, e.g., replacing peptone with chicken feather hydrolysate, has been tested many times (Alamnie et al. 2024; Branska et al. 2020). Protein waste hydrolysate is primarily a source of nitrogen in microbial cultivation, but can also serve as a source of carbon.

This study is focused on the upcycling of waste derived from the production of meat products and spent brewer’s yeast. All valorized waste was rich in protein. In dry matter, chicken feather contains up to 90% of proteins (Stiborova et al. 2016), chicken or fish leftover can contain up to 63 or 58% proteins, respectively (Nirmal et al. 2022; Talha et al. 2024) and spent brewer`s yeast contain up to 45% proteins (Vélez-Erazo et al. 2021). The selected method of valorization of these kinds of waste was their hydrolysis and subsequent use of the hydrolysates as a substrate for microbial growth. When hyper-ammonia producing (HAP) bacteria are grown in the medium containing protein-rich organic substrate, ammonium ions are released into the environment. These HAP bacteria are mainly found among anaerobes in the rumen of ruminants (Ward et al. 2018), in fermented animal products (Zhao & Eun, 2018) but also among facultatively anaerobic bacteria, such as *Bacillus subtilis* (Choi et al. 2014) or *Escherichia coli* (Tatemichi et al. 2020). To date, the highest ammonium ion concentration achieved from protein waste using microorganisms was 3.34 g/L, obtained from okara (soy milk production waste) using modified *Saccharomyces cerevisiae* yeast (Watanabe et al. 2020). However, even lower concentrations of ammonium ions, in the order of hundreds of mg, are advantageous for the production of biofertilizers (Ward et al. 2018).

This paper aims to test variously produced hydrolysates of animal waste products and spent brewer’s yeast for the release of ammonium ions. For this purpose, the bacterium *Pantoea agglomerans* DBM 3797, a hop endophyte, was used, which had already been found in previous research to release ammonium ions when grown in LB medium and which exhibits features of an organism with plant growth-promoting (PGP) characteristics (Patakova et al. 2024). As a proof of concept, i.e. for the potential development of biofertilizer and plant growth promoting agent in one, a selected feather hydrolysate with *P. agglomerans* was tested as a potential substitute for Murashige and Skoog Basal Salt Mixture, (a plant tissue culture growth medium), for the growth of 3-week-old lettuce (*Lactuca sativa* var. *Longifolia*) seedlings.

## Experimental

All chemicals were purchased from Merck (Germany) if not stated otherwise.

### Characterization of food waste materials

Chicken feathers (feathers) and frozen leftovers after separation of chicken meat (chicken leftovers) were obtained from Rabbit a. s., Trhovy Stepanov, Czechia (age of chicken 36 days). Spent brewer’s yeast was supplied by the Brewery of the Department of Biotechnology, UCT Prague. Carp residues from Trebon carp, especially skin, scales, guts, bones, were received from Lahovice fishponds, Czechia. All materials were used without further modification, except for the feathers, which had been dried for feather alkaline hydrolysate production and ground to a size of up to 10 mm for feather acid hydrolysate production.

## Hydrolysates production

Hydrolysates production steps are shown in Table 1.

**Table 1:** Overview of the hydrolysates preparation

Hydrolysate Type	Label	Weight of Waste Material	Chemical Additions	Treatment Steps
Chicken leftover acid hydrolysate 1	A	140 g	1 L water 10 g malic acid	1) Autoclave: 60 min, 121 °C 2) Tempered shaker: 24 h, 80 °C, 150 rpm 3) Filter paper filtration 4) Centrifugation: 10 min, 6000 rpm
Chicken leftover enzyme hydrolysate	B	100 g	0.9 L water 1 mL papain enzyme (55 g/L)	1) Autoclave: 15 min, 121 °C 2) Enzyme addition 3) Tempered shaker: 24 h, 80 °C, 150 rpm 4) Büchner funnel filtration 5) Centrifugation: 10 min, 6000 rpm
Chicken leftover acid hydrolysate 2	C	2000 g	15 L water 100 g malic acid	1) Autoclave: 5 h, 140 °C 2) Suction nutch filter filtration
Feather acid hydrolysate	D	2000 g	15 L water 100 g malic acid	1) Autoclave: 5 h, 140 °C 2) Suction nutch filter filtration
Carp residues acid hydrolysate	E	2000 g	15 L water 100 g malic acid	1) Autoclave: 5 h, 140 °C 2) Suction nutch filter filtration
Feather alkaline hydrolysate	F	20 g	1 L water 6 g potassium hydroxide	1) Tempered shaker: 24 h, 80 °C, 150 rpm 2) Büchner funnel filtration 3) Centrifugation: 10 min, 6000 rpm 4) Büchner funnel filtration
Spent brewer’s yeast autolysate	G	500 g	0.5 L water	1) Tempered shaker: 24 h, 55 °C, 150 rpm 2) Filter paper filtration 3) Büchner funnel filtration

For the following experiments, only the liquid fraction of all hydrolysates (after filtration or centrifugation or both) was used. All hydrolysates were adjusted to pH 7 and sterilized by filtration through a PVDF filter with 0.22 μm pores except for chicken leftover enzyme hydrolysate (B) and feather alkaline hydrolysate (F), which were sterilized in an autoclave without clarification. The filtration of hydrolysates B and F was performed only for specific analyses (optical density measurement and determination of ammonium ions content). The produced sterile hydrolysates were used for bacterial cultivations or stored in a refrigerator at 6–8 °C.

## Characterisation of hydrolysates

### Matrix-assisted laser desorption/ionization - time of flight mass spectrometry (MALDI-TOF MS)

The analysis of the hydrolysates by MALDI-TOF MS was performed to determine the approximate peptide content, especially the peptide length. The samples were desalted and concentrated using ZipTips^®^ with C18 chromatography medium (Millipore Corporation, USA). The elution volume was 10 µL (Smirnova et al. 2021). An aliquot of the eluted sample after ZipTip purification was mixed with HCCA (α-cyano-4-hydroxycinnamic acid,) solution (10 mg of HCCA in 1 mL of a mixture of 50% ACN and 0.1% TFA) in a 1:1 (v/v) ratio. Then, 1 µL of the matrix-mixed sample or calibration standard (Bruker Daltonics, Germany) was spotted in triplicate and left to dry on three spots of a MALDI steel target. Measurements were performed using an Autoflex Speed MALDI-TOF mass spectrometer (Bruker Daltonics, Germany). Analyses were carried out in positive reflector mode using a Nd: YAG laser operating at 355 nm. Spectra were acquired using FlexControl (Bruker Daltonics, Germany) in a mass range of 380–5000 *m/z*. The resulting spectra were processed with mMass version 5.5.0 (Czech Academy of Sciences). The spectra were cropped to 3000 *m/z* and normalized using the “Normalize Intensities” function in mMass, which rescales all signal intensities relative to the most intense peak (assigned a value of 100%), while preserving the relative intensity ratios of all other signals.

### Determination of carbon, nitrogen, and sulfur content

At first, frozen samples of 5 mL hydrolysates were lyophilised using the lyophiliser Heteo PowerDry LL 3000 (Thermo Fisher Scientific, Germany). The carbon, nitrogen, and sulfur contents were determined using the Elementar Vario Cube device (Elementar, Germany). The lyophilised samples weighing 2–3 mg, were burned in a stream of oxygen at a temperature up to 1200 °C. The resulting combustion products were then detected by thermal conductivity detector or infrared detector, and results were expressed as a percentage of the total sample content. The results of the analysis consisted of all types of combusted carbon and sulfur, i.e., of both inorganic and organic origins. The precision of the method was determined by simultaneous analysis of 5 mg of 4-aminobenzenesulfonic acid as a standard in the modules of carbon, hydrogen, nitrogen, and sulfur to < 0.1% for each element.

## Microbial culture

### Bacterial inoculum

*Pantoea agglomerans* DBM 3797 stock cultures were maintained at −80 °C in 30% glycerol. Bacteria were reactivated from cryopreserved glycerol stocks in fresh lysogenic broth (LB) containing [g/L]: Tryptone 10, Yeast extract 5, NaCl 5 (Penta, Czechia). The cultivation was performed in 250 mL Erlenmeyer flasks with 100 mL of medium placed on a rotary shaker under the following conditions: 30 °C, 150 rpm, 24 h. The culture was used as a 1% v/v inoculum for microbial cultivation in the hydrolysates adjusted to pH 7 or in fresh LB medium (control experiment).

### Bacterial growth

The *Pantoea agglomerans* bacteria were cultivated in hydrolysates or LB medium in microwell plates that were placed in the Bioscreen C device (Oy Growth Curves Ab Ltd., Finland) to obtain growth curves by measuring the optical density every 30 min for 22 h at a set temperature of 30 °C and shaking (1 min, linear shaking, speed: normal, amplitude: medium) before and after each OD measurement. The volume of 200 µL of medium was inoculated with 5 µL of inoculum with the optical density set to 0.1. Each experiment was performed in quintuplicate, but only chicken leftover enzyme hydrolysate (B) in triplicate. Lag and log phase durations were determined from growth curves in MS Excel (Microsoft, USA). For pH value measurement and determination of ammonium ions content, the cultivations were carried out in 100 mL Erlenmeyer flasks with 50 mL of medium inoculated with 1% v/v inoculum and a temperature of 30 °C, with agitation speed set at 150 rpm for 48 h. Cultivation experiments in flasks were done in triplicate.

### Calculation of specific growth rate

The specific growth rate of *Pantoea agglomerans* DBM 3797 in different cultivation media was calculated according to the Eq. (1).

$\:{\mu\:}_{x}=\frac{{ln}_{{OD}_{t2}}-{ln}_{{OD}_{t1}}}{{t}_{2}-{t}_{1}}\:\left({h}^{-1}\right)$It is the rate calculated from the starting OD at time t_1_, corresponding to the start of the exponential phase and OD at time t_2_, corresponding to the end of the exponential phase.

The plots of optical density versus time were converted to logarithmic plots to find the linear region of the exponential phase. The coefficient of linearity (R^2^) was > 0.98 in all cases, with the exception for chicken acid hydrolysate (A), where it was > 0.91.

## Determination of ammonium ions content

The concentration of ammonium ions in the samples obtained after microbial cultivation was determined using an ammonium selective electrode (Hanna^®^, USA) set up and calibrated according to the manufacturer’s instructions. The device was prepared for measurement by three-point calibration, namely 0.001, 0.01, and 0.1 mol/L of ammonium ion standard (Hanna^®^, USA). The volume of the sample supernatant (centrifuged for 5 min at 6000 rpm using centrifuge Hettich, Schoeller, Germany) was set at 25 mL per determination. For the measurement, 0.5 mL of ISA standard (Hanna^®^, USA) was added to the glass sample vial. The ammonia concentration was measured with an ion-selective electrode inserted into the stirred solution. The concentration was evaluated in mol/L of ammonium ions, which were converted to g/L of ammonium ions in the sample.

## *P. agglomerans* colony-forming unit count test

The colony-forming unit (CFU) count test was performed to determine the concentration of *Pantoea agglomerans* grown in the alkaline feather hydrolysate, and used in lettuce growth tests. The culture was first serially diluted in saline solution using 10-fold dilution in each step to reach a dilution factor of 10^6^. Petri dishes containing LB agar medium were inoculated with 100 µL of diluted culture, and placed in a thermostat set at 30 °C for 24 h. The number of colonies grown on agar were counted and expressed as CFU/mL using a simple formula:

$$\:\frac{\text{C}\text{F}\text{U}}{\text{m}\text{L}}=\frac{No.\:of\:colonies*Total\:dilution\:factor}{Volume\:of\:culture\:plated\:in\:mL}$$

## Testing of lettuce (*Lactuca sativa* var. *Longifolia*) growth

Seedlings of romaine lettuce *Lactuca sativa* var. *Longifolia*, 3-week-old plants planted in soil with sand, were purchased from the market garden OBI (Czechia). Seedlings were grown in a Secret Jardin Dark Propagator 60 × 40 × 60 cm greenhouse tent (Growmarket, Czechia) with simulated 12 h daylight (lighting by GENT G-LED 26 W, 6500 K placed in the middle of the tent ceiling). The seedlings were grown for one week at a temperature of 26–28 °C and humidity of 70–80%, see Supplement Fig. 2. Watering was carried out on the first and fourth day. The first group of seedlings was watered with Murashige-Skoog Basal Salt Mixture medium (MS medium, Sigma Aldrich, Germany), the second group with the feather hydrolysate medium in which *P. agglomerans* DBM 3797 was cultured for 2 days, and the third group was watered with distilled water. The feather hydrolysate with *Pantoea* agglomerans was diluted with distilled water in a ratio of 1:20 before watering, and the concentration of *P. agglomerans* DBM 3797 in diluted hydrolysate was 4.6 ± 0.7 × 10^8^ CFU/mL. The approximate concentration of nitrogen (occurring in the forms of amino acids, peptides and ammonium ions) in the diluted feather alkaline hydrolysate, was 0.1 g/L, calculated from elemental analysis and considering the dilution (i.e. 9.9% of total nitrogen from 20 g feathers/L according to Tables 1 and 3 for feather alkaline hydrolysate, respectively, divided by a dilution factor of 20). The MS medium, contained approximately 0.84 g/L nitrogen (in forms of ammonium ions and nitrates) calculated according to the composition specified by the manufacturer. The volume of watering was 10 mL/seedling and the watering medium was injected into the soil by syringe. Each pack of seedlings included 6 individual seedlings that were watered independently with the same agent. Quantification of the effect of each watering agent on plant growth was done by weighing the fresh seedlings carefully extracted from soil, washed with water and drained, measuring the length of their roots and also counting individual leaves. After processing of fresh seedlings, they were dried in an oven at a temperature of 70 °C until a constant weight and dry lettuce mass of each seedling was determined.

## Statistical analyses

One-way ANOVA for the ammonia ion concentration, *Pantoea agglomerans* growth and lettuce growth experiments, were evaluated using IBM SPSS Statistics, Version 31.0.1.0 (49) software (IBM, USA) with a set α value lower than 0.05. This value was considered statistically significant for ANOVA test. The data obtained from the one-way ANOVA test was then corrected using post-hoc Duncan’s multiple range test, to compare the values of specific parameters (Lag Phase, Log Phase, Specific Growth Rate, OD, pH, Ammonium Ions Concentration, Ammonium Ions Concentration per Gram of Feedstock, Fresh Lettuce Mass, Dry Lettuce Mass, Number of Leaves, Root Length) of the specific groups (hydrolysates or watering agents) at a significance level of *p* < 0.05. As an output, there were obtained statistically significant changes among the samples. The specific numerical data are shown in Supplementary Tables 1, 2 and 3. The chart in Fig. 2 was created using MS Excel Software (Microsoft, USA).

## Results

To compare the growth in LB culture medium (Control) and in the hydrolysates, *Pantoea agglomerans* DBM 3797 was cultivated in a microwell plate in the Bioscreen C device for 22 h, see Table 2. After 22 h, cultures in all cases reached a stationary growth phase, i.e., OD values in all wells were stable in the last 4 h of measurement. Lag phase in feather alkaline hydrolysate (F) was comparable to that in the control. Interestingly, growth in spent brewer’s yeast autolysate (G) required a long adaptation of the bacteria (lag phase 5.8 h), but reached an optical density comparable to the control. The highest specific growth rate using the hydrolysates, 0.63 h^− 1^, was achieved in carp residue acid hydrolysate (E). In contrast, the lowest specific growth rate, 0.11 h^− 1^, was achieved in feather acid hydrolysate (D). Unfortunately, in some cases, optical density values above 1 were reached during growth, where the linear relationship between light absorbance and particle concentration was not guaranteed. This may have biased the results of specific growth rates and maximum optical densities for LB culture medium (Control), feather acid hydrolysate (D) and spent brewer’s yeast autolysate (G).

**Table 2:** Comparison of the *Pantoea agglomerans* DBM 3797 growth in LB medium and in hydrolysates

Hydrolysate Type	Label	Lag Phase [h]	Log Phase [h]	Specific Growth Rate [h^− 1^]	OD_22 hours_
LB medium	Control	3.60 ± 0.20^a^	9.20 ± 0.40^c^	0.27 ± 0.01^cde^	1.36 ± 0.01^g^
Chicken leftover acid hydrolysate 1	A	6.50 ± 0.63^d^	9.10 ± 0.92^bc^	0.31 ± 0.04^e^	0.46 ± 0.02^a^
Chicken leftover enzyme hydrolysate	B	5.33 ± 0.24^b^	5.50 ± 1.01^a^	0.28 ± 0.01^de^	0.89 ± 0.01^d^
Chicken leftover acid hydrolysate 2	C	6.50 ± 0.32^d^	11.20 ± 0.68^d^	0.22 ± 0.05^bc^	0.99 ± 0.03^e^
Feather acid hydrolysate	D	5.10 ± 0.20^b^	5.20 ± 0.24^a^	0.11 ± 0.01^a^	1.28 ± 0.02^f^
Carp residues acid hydrolysate	E	5.50 ± 0.00^bc^	6.10 ± 0.58^a^	0.63 ± 0.06^f^	0.74 ± 0.02^c^
Feather alkaline hydrolysate	F	3.30 ± 0.24^a^	7.90 ± 0.20^b^	0.19 ± 0.01^b^	0.56 ± 0.01^b^
Spent brewer’s yeast autolysate	G	5.80 ± 0.24^c^	8.20 ± 0.68^bc^	0.25 ± 0.01^cd^	1.25 ± 0.02^f^

The letters refer to statistical analysis correlated with duncan’s multiple range test for each parameter separately. the values followed by the same letter are not significantly different from one another at significance level *p* < 0.05. Conversely, values that do not share the same letter are significantly different. the letter (a) represents the lowest mean value of all tested groups for a specific parameter And the letters (f/g) represent the highest mean value. the results of duncan’s test for each parameter (Lag Phase, log Phase, specific growth rate And OD) can be seen in supplement Table 1a, b, c, and d

One-way ANOVA test followed by Duncan`s post-hoc test were performed to find out statistically significant differences in *P. agglomerans* DBM 3797 growth parameters. Bacterial specific growth rate did not differ statistically significantly from the growth in LB medium (control) in cases of hydrolysates (A, B, C and G). Length of lag phase, i.e., the adaptation phase to the cultivation environment, was always significantly longer in all types of hydrolysate compared to LB medium, except for feather alkaline hydrolysate (F). The exponential growth phase was significantly shorter compared to the control for hydrolysates (B, D, E, and F), significantly longer for hydrolysate (C), and comparable in other cases. Spent brewer’s yeast hydrolysate (G) did not differ significantly from the control in two growth parameters, i.e. log phase length and specific growth rate.

Despite the different growth dynamics, the ability of the culture to grow in hydrolysates was clearly demonstrated.

Another (next) experiment focused on evaluating the amount of ammonium ions released into the medium. Supernatants of the cultivation media after 48 h (after reaching the stationary phase of growth) were analyzed using the ISE electrode and the final ammonia concentrations are given in g/L and also recalculated per 1 g of hydrolysed feedstock (in the case of LB medium, the ammonia concentration was recalculated per g of tryptone and yeast extract). The conversion of the ammonium ion concentration per gram of the original material prior to hydrolysis shows which material (waste), in combination with a particular type of hydrolysis, had the greatest potential for ammonium ion release. In addition, nitrogen, carbon and sulfur contents of the hydrolysates are given in the Table 3 to enable their mutual comparison.

**Table 3:** Release of ammonium ions from LB medium and different hydrolysates

Hydrolysate Type	Label	pH		c_NH4+_ (g/L)		c_NH4+_ (mg/g)		Total *N* (%)		Total C (%)		Total S (%)	
LB medium	Control	8.64 ± 0.04^e^	0.216 ± 0.008^a^		14.40 ± 0.53^e^		NT		NT		NT		
Chicken leftover acid hydrolysate 1	A	8.37 ± 0.02^b^	0.263 ± 0.019^b^		1.88 ± 0.14^a^		8.8		35.5		0.4		
Chicken leftover enzyme hydrolysate	B	7.95 ± 0.02^a^	0.774 ± 0.018^e^		7.74 ± 0.18^d^		NT		NT		NT		
Chicken leftover acid hydrolysate 2	C	8.45 ± 0.03^c^	0.707 ± 0.015^d^		5.32 ± 0.11^c^		11.3		37.5		0.7		
Feather acid hydrolysate	D	8.56 ± 0.02^d^	0.943 ± 0.001^g^		7.09 ± 0.01^d^		12.3		43.5		1.6		
Carp residues acid hydrolysate	E	8.59 ± 0.02^de^	0.502 ± 0.005^c^		3.77 ± 0.04^b^		8.8		41.5		0.7		
Feather alkaline hydrolysate	F	9.03 ± 0.02^f^	0.824 ± 0.035^f^		41.20 ± 1.75^f^		9.9		34.9		2.0		
Spent brewer’s yeast autolysate	G	8.63 ± 0.02^e^	0.829 ± 0.044^f^		1.66 ± 0.09^a^		11.9		40.7		0.7		

The letters refer to statistical analysis correlated with duncan’s multiple range test for each parameter separately. the values followed by the same letter are not significantly different from one another at significance level *p* < 0.05. Conversely, values that do not share the same letter are significantly different. the letter (a) represents the lowest mean value of all tested groups for a specific parameter and the letters (f/g) represent the highest mean value. the results of duncan’s test for each parameter (pH, ammonium ions concentration and ammonium ions concentration per gram of Feedstock) can be seen in supplement Table 2a, b, c

The best results were observed for feather hydrolysates produced by acid/alkali hydrolysis and for spent brewer’s yeast autolysate, where the concentration of ammonium ions exceeded 0.8 g per liter. The highest ammonium ion concentration was 0.943 g/L for feather acid hydrolysate (D). The lowest concentration of ammonium ions (0.2 g/L) in the hydrolysates tested was found in the chicken leftover acid hydrolysate (A) using a tempered shaker. When the concentration of released ammonium ions was recalculated per gram of hydrolysate feedstock, the outstanding result was achieved for the feather alkaline hydrolysate (F). However, it cannot be concluded that the good values obtained from the growth curve measurements (short lag phase, high specific growth rate and high final optical densities given in Table 2) were also indicators of good results for the release of ammonium ions. The release of ammonia ions into cultivation medium as well as concentration of ammonium ions per gram of protein material differed statistically significantly from that obtained after the cultivation in LB medium in all cases.

**Fig. 1:**
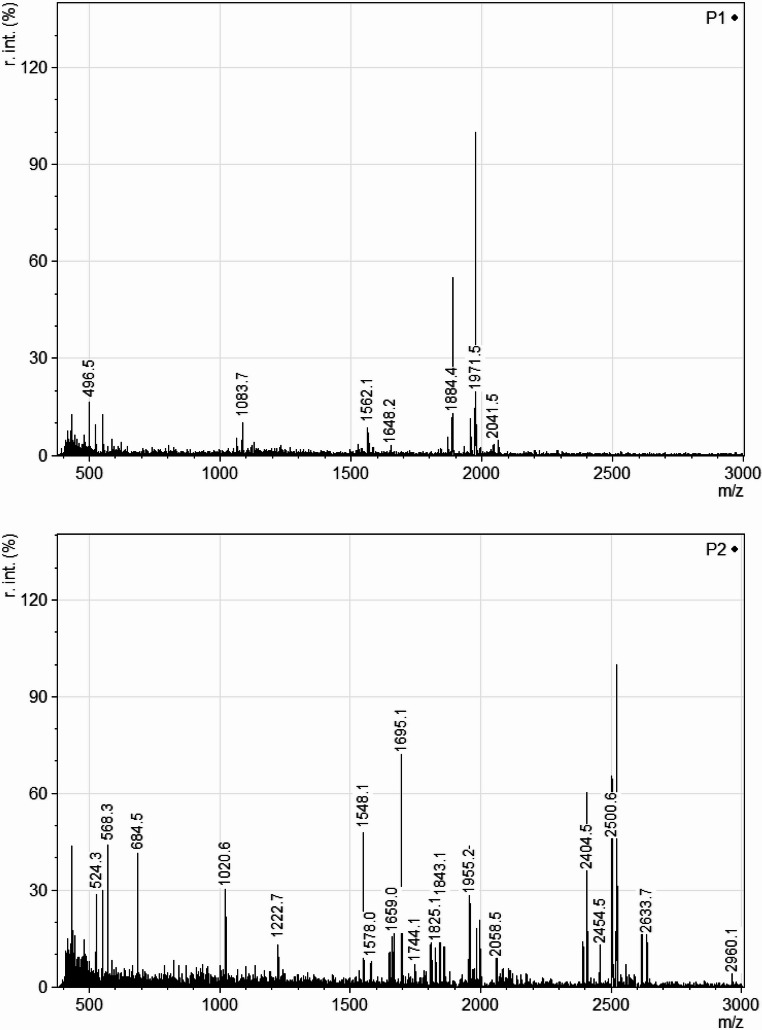
MALDI-TOF mass spectra representing the sample of feather alkaline hydrolysate (first picture – P1) and feather acid hydrolysate (second picture – P2)

Nitrogen content of the hydrolysates (Table 3) ranged from 8.8 to 12.3% and showed no significant change between hydrolysates. This is the reason why the MALDI-TOF MS assay, was done to show the size distribution of soluble peptides in the material, see Table 4; Fig. 1 and Supplement Fig. 1. The peptide distribution is expressed in 5 groups according to the peptide mass per charge (*m/z*) number ratio, ranging from 400 to 2000, which is equal to sizes expressed in Daltons. The numbers in Table 4 represent different types of fragments and are not related to their frequency of occurrence. The greatest abundance of peptides was observed in the first group, with a range of values from 400 to 800 Da. Most of the heavier/longer peptides with *m/z* ratios from 1200 to more than 2000 Da were observed in the feather hydrolysates, as is shown in Fig. 1. When acids or bases were used for hydrolysis, the peptides were lighter/shorter compared to other hydrolysates. The hydrolysates after cultivation with *Pantoea agglomerans* DBM 3797 were also analyzed to identify changes in the peptide content of the hydrolysates; data shown in Supplement Fig. 1. The most significant changes were seen in the samples of meat and feather hydrolysates. The bacteria were able to completely digest longer peptides or convert them to smaller ones, as no longer peptides were detected after the cultivation.

**Table 4:** MALDI-TOF MS analysis of soluble peptides in LB medium and in the different hydrolysates

		Amount of Peptide Types in Selected Range of Dalton Sizes:				
**Hydrolysate Type**	**Label**	400–800	801–1200	1201–1600	1601–2000	> 2001
LB medium	Control	7	7	ND	ND	ND
Chicken leftover acid hydrolysate	A	23	ND	ND	ND	ND
after cultivation with *P. agglomerans* DBM 3797		6	ND	ND	4	4
Chicken leftover enzyme hydrolysate	B	6	1	ND	1	ND
after cultivation with *P. agglomerans* DBM 3797		5	6	2	1	ND
Chicken leftover acid hydrolysate 2	C	4	ND	ND	4	2
after cultivation with *P. agglomerans* DBM 3797		3	ND	ND	1	1
Feather acid hydrolysate	D	4	1	3	13	8
after cultivation with *P. agglomerans* DBM 3797		9	5	1	ND	ND
Carp residues acid hydrolysate	E	13	5	1	1	3
after cultivation with *P. agglomerans* DBM 3797		2	1	ND	2	ND
Feather alkaline hydrolysate	F	2	2	2	5	2
Spent brewer’s yeast autolysate	G	11	3	2	ND	ND

**Fig. 2:**
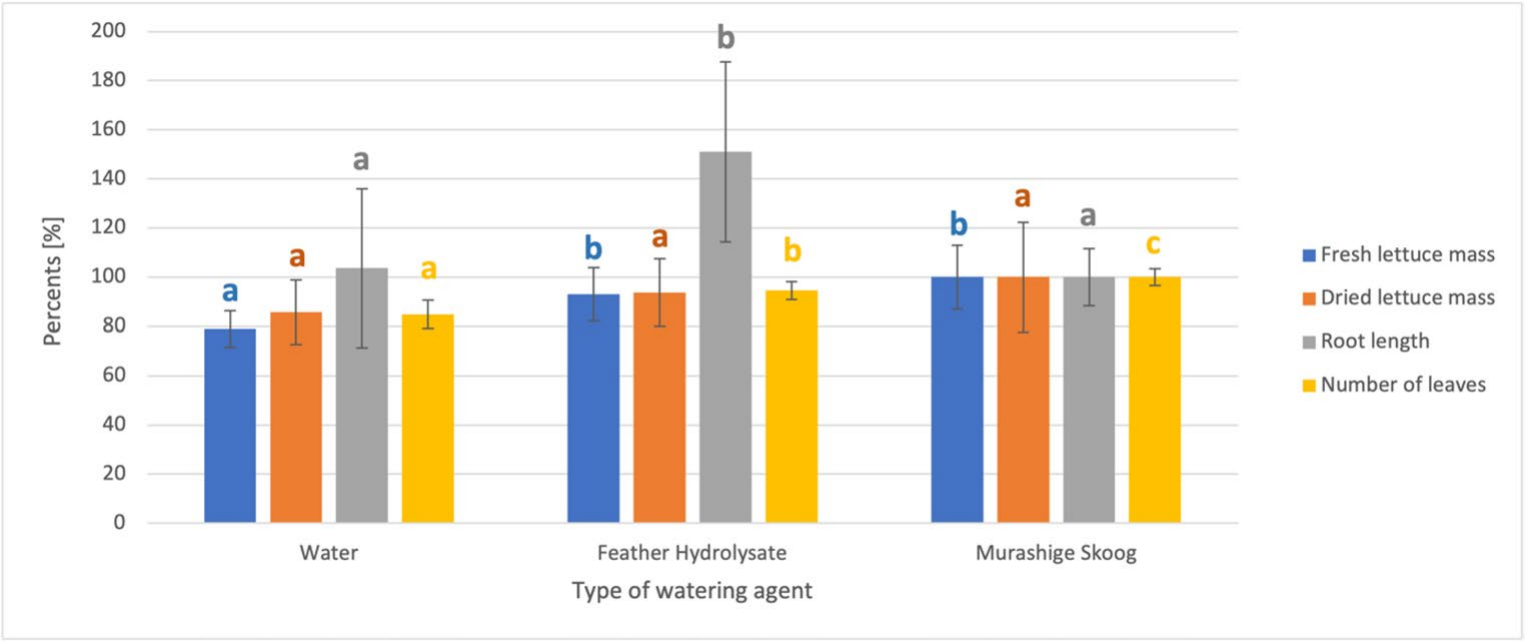
Comparison of the growth parameters of lettuce watered with different agents: water, Murashige-Skoog Basal salt mixture medium and feather hydrolysate. The letters refer to statistical analysis correlated with Duncan’s multiple range test for each parameter separately. Murashige Skoog medium was taken as a control. The values followed by the same letter of a specific color are not significantly different from one another at significance level *p* < 0.05. Conversely, values that do not share the same letter are significantly different. The letter (**a**) represents the lowest mean value of all tested groups for a specific parameter and the letters (**b**/**c**) represent the highest mean value. The results of Duncan’s test for each parameter (Fresh lettuce mass, dry lettuce mass, number of leaves, root length) can be seen in Supplement Table 3

To demonstrate the potential use of hydrolysate with *P. agglomerans* bacteria as a possible biofertilizer and plant growth promoter, we conducted a short experiment with lettuce seedlings, watered by three agents: Murashige-Skoog Basal Salt Mixture medium (MS); hydrolysate with *P. agglomerans* (F) and distilled water, see Fig. 2, Supplement Fig. 2 and Supplement Table 3. The feather alkaline hydrolysate was chosen because it exhibited the highest ammonia ions release calculated per gram of feedstock, feathers. The application of feather alkaline hydrolysate with cultivated endophytic bacteria *P. agglomerans* DBM 3797 to the soil led in particular to the promotion of root lengths, which were more than 40% longer compared to use of other watering agents as is shown in Fig. 2. Photographs comparing root lengths are shown in Supplement Fig. 3.

A one-way ANOVA followed by Duncan`s post-hoc test were done separately for individual growth parameters, i.e., fresh lettuce mass, dry lettuce mass, root length and number of leaves. The feather hydrolysate applied with *P. agglomerans* outperformed both MS medium and distilled water in root length. The results obtained when watering with MS medium and the aforementioned hydrolysate showed no statistically significant differences in the values of fresh and dry lettuce mass. To obtain a more conclusive result, it would be necessary to conduct a larger number of experiments and probably to extend the plant cultivation period.

## Discussion

*Pantoea agglomerans* DBM 3797 exceeded in ammonium ion production (up to 0.9 g/L) other HAP non-modified bacteria described so far, e.g. *Bacillus subtilis* 168 (0.45 g/L) (Choi et al. 2014), *Clostridium aminophilum* (0.16 g/L) (Ward et al. 2018) or *E. coli* (0.325 g/L) (Tatemichi et al. 2020). To achieve higher concentrations comparable to our isolate, the HAP strains had to be genetically modified. These modifications targeted reuptake of ammonia by HAP bacteria and its use for glutamate biosynthesis (Tatemichi et al. 2020). However, the use of genetically manipulated bacteria is practically banned in Europe in agriculture and food processing. The use of a wild endophyte strain in a plant growth promoter or a biofertilizer is far less problematic. In addition, the genome of the *P. agglomerans* DBM 3797 strain has already been sequenced (*Pantoea agglomerans* genome, 2021), and its potential for agricultural applications such as the production of indole acetic acid derivatives, siderophores, or gluconic acid has been described (Patakova et al. 2024). *P. agglomerans* is a controversial bacterial species with two faces (Dutkiewicz et al. 2016). On the one hand, some strains have already been approved for biological plant protection (Duchateau et al. 2024), but on the other hand, it is an opportunistic pathogen (Kaur et al. 2020). A detailed analysis of various features, supported by genome sequence analysis, did not reveal any significant pathogenic potential of the strain and classified it into a group of probably harmless strains isolated from the above-ground parts of plants (Patakova et al. 2024). However, a detailed assessment of all risks associated with this particular strain is a matter for future research.

The feather alkaline hydrolysate (F) was a very good substrate for the release of ammonium ions into the medium. The short lag time of the growth curve in this hydrolysate indicates that this hydrolysate does not contain inhibitory substances or contains peptides readily degradable by constitutively formed enzymes of *P. agglomerans* DBM 3797. In addition, the highest concentration of released ammonium ions per gram of original material (41 mg/g) was achieved in this hydrolysate. The same produced hydrolysate has already been used as a nitrogen source for the butanol-producing bacteria *Clostridium beijerinckii* (Branska et al. 2020), lactic acid producers, *Lactobacillus reuteri* (Gharwalova et al., 2018) and *Lactobacillus casei* (Paulova et al. 2020) or *E. coli* growth (Stiborova et al. 2016). Nevertheless, the feather acid hydrolysate (D) was also an excellent *P. agglomerans* substrate. The advantage of feather acid hydrolysate is its environmentally friendly preparation using malic acid instead of mineral acid (Solcova et al. 2021), and the fact that the hydrolysate has already been tested as a biofertilizer for cayenne pepper (Vavrova et al. 2022). Hydrolysates of chicken leftovers or fish wastes were confirmed to be convenient for *P. agglomerans* growth, which is in accordance with the previously published results (Nakamura et al. 2021; Kolek et al. 2023). The spent brewer’s yeast has already been tested as a fermentation substrate for lactic acid production (Radosavljević et al. 2020) and verified to be an excellent substrate for ammonium ion release in this study.

The release of ammonium ions in different hydrolysates could also be affected by different amino acid profiles. While the most abundant amino acids in chicken feathers were serine, glutamic acid, glycine, leucine, proline, alanine, and valine but also hydrophobic acids such as tryptophan (Rajabi et al. 2020), in yeast extract, these were glutamic acid, aspartic acid, lysine and leucine (Tomé 2021), and in casein (tryptone in LB medium) they included glutamic and aspartic acids, threonine, lysine, leucine and others (Matsunaga et al. 2018). For human fecal bacteria growing on peptides and amino acids (Richardson et al. 2013), preferential utilization and ammonium ion release was found in the case of aspartic acid, serine, lysine, and glutamic acid.

Both feather hydrolysates (alkaline and acid) contained longer peptides with *m/z* ratios from 1200 to more than 2000 Da, which were probably better utilized than shorter peptides. Unfortunately, for the species *Pantoea agglomerans*, growth on soluble peptides is not a sufficiently studied discipline. However, a model of milk casein degradation is available for *Lactococcus lactis* (Savijoki et al. 2006), which clearly shows that extracellular proteases preferentially attack proteins of larger sizes, breaking them into smaller pieces that are transported by oligopeptide transporters into cells, where they are cleaved by peptidases into amino acids. Within the *Pantoea agglomerans* DBM 3797 chromosome, there can be found genes coding for proteases, oligopeptidase and Opp permease transporters (for oligopeptide transport), therefore it seems likely that peptide-mediated growth may work similarly. On the contrary, small peptides from meat produced by acid hydrolysis, which resulted in unsatisfactory *P. agglomerans* growth, might have antimicrobial activity; this has been reported previously for meat peptides (López-García et al. 2022; López-Pedrouso et al. 2023). In addition, MALDI-TOF MS assay has already been used for identification of different metabolic patterns of bacteria (Cejnar et al. 2018). When all types of chicken meat hydrolysates (A, B, C) were used, a new peak at 1889 *m/z* was detected in the culture medium. This peak could correspond to protease or another secreted protein.

Since the nitrogen content in diluted feather hydrolysate with *P. agglomerans* (approximately 0.1 g/L) was significantly lower than in MS medium (0.84 g/L), it seems that promotion of lettuce growth by the hydrolysate cannot be explained only by nitrogen content, but also by other compounds probably present in the hydrolysate, such as indole-3-acetic acid derivatives formed from tryptophan by *P. agglomerans*. The formation of these growth stimulators was already confirmed by this bacterial strain (Patakova et al. 2024). In addition to the production of indole-acetic acid, peptides from chicken feathers may also have a supportive effect. Both effects (indole-acetic acid production and presence of specific peptides derived from feather) have been confirmed in previous experiments with beans (Devi et al. 2024) or rice (Sahoo et al. 2023). Furthermore, growth promotion after the addition of a combined preparation (hydrolysate and endophytic bacteria) may consist in the suppression of phytopathogenic fungi growth, as observed in the study by Jeong et al. (2010).

Plant growth promotion induced by the application of animal hydrolysates in general has already been described (Costa et al. 2024) and it seems that their main effect was to promote the growth of microorganisms in the rhizosphere, which in turn leads to better plant growth. When animal hydrolysates were applied to the soil, the effect on root elongation in corn (Santi et al. 2017) or tomato (Casadesús et al. 2020) was particularly noted. In case of lettuce, the root elongation was described in hydroponic culture (Xiang et al. 2025). In addition to the stimulating effects already mentioned, hydrolysates can contribute to easier uptake of elements such as K, Zn, Cu, and Mn from the environment (Santi et al. 2017). However, when applying animal hydrolysates, it is necessary to take into account the dosage (appropriate dilution) in order to avoid triggering a stress response in the treated plants (Rouphael et al. 2021). Although the joint application of animal hydrolysates and endophytic microorganisms in two separate portions was tested (Nafady et al. 2018), no preparation has yet been tested in which animal hydrolysate was used as a culture medium for endophytes and, after cultivation, this medium was used with live cells as a biofertilizer and plant stimulant in one.

## Conclusion

The study indicates the new option how to valorize protein hydrolysate made from agriculture and food industry waste as a cultivation medium for endophytes. Endophytic bacteria, i.e., bacteria isolated from the internal environment of plants, can help plants combat various types of stress. Protein waste hydrolysates have a similar effect, but achieved in a different way (through bioactive peptides). After cultivation of endophytic bacteria, such as *P. agglomerans*, this spent cultivation medium, containing living cells, peptides, ammonium ions and potentially also plant growth promoting compounds, might be used as a bio-fertilizer and plant bio-stimulant in one. The proof of concept experiment in which feather alkaline hydrolysate, cheap for preparation, together with live bacterial cells of *P. agglomerans* DBM 3797 was applied to soil for growth promotion of lettuce seedlings, confirmed that the developed agent might function. In the future, large-scale and long-term research will need to be conducted in agriculture to test different crops, application dose and time and to evaluate more plant factors to better understand and confirm the efficacy of the described bio-stimulant and fertilizer agent. After extensive field tests, this could be a way to reduce chemical fertilizer with bio-based alternatives, including both microbial biostimulants and biofertilizers, or their combination.

## Supplementary Information

Below is the link to the electronic supplementary material.

Supplementary Material 1 (DOCX 7.09 MB)

## Data Availability

All data supporting the findings of this study are available within the paper and its Supplementary Information.

## References

[CR1] Alamnie G, Melake A, Berhanu Y, Alemu M, Damtew B, Aemiro A (2024) Chicken feather protein hydrolysate as a low cost peptone source for microbial cultivation: a promising perspective of economic and environmental advantage. Case Stud Chem Environ Eng 9:100741. 10.1016/j.cscee.2024.100741

[CR2] Bernhard A (2010) The Nitrogen Cycle: Processes, Players, and Human Impact. Nat Educ Knowl. https://www.nature.com/scitable/knowledge/library/the-nitrogen-cycle-processes-players-and-human-15644632/ Accessed 20 June 20 2025

[CR3] Branska B, Fortova L, Dvorakova M, Liu H, Patakova P, Zhang J, Melzoch K (2020) Chicken feather and wheat straw hydrolysate for direct utilization in biobutanol production. Renew Energy 145:1941–1948. 10.1016/j.renene.2019.07.094

[CR4] Casadesús A, Pérez-Llorca M, Munné-Bosch S, Polo J (2020) An enzymatically hydrolyzed animal protein-based biostimulant (Pepton) increases salicylic acid and promotes growth of tomato roots under temperature and nutrient stress. Front Plant Sci 11:953. 10.3389/fpls.2020.00953 32714352 10.3389/fpls.2020.00953 PMC7342040

[CR5] Cejnar P, Kuckova S, Prochazka A, Karamonova L, Svobodova B (2018) Principal component analysis of normalized full spectrum mass spectrometry data in multiMS-toolbox: an effective tool to identify important factors for classification of different metabolic patterns and bacterial strains. Rapid Commun Mass Spectrom 32(11):871–881. 10.1002/rcm.8110 29520858 10.1002/rcm.8110

[CR6] Chen J, Li J, Li W, Li P, Zhu R, Zhong Y, Zhang W, Li T (2024) The optimal ammonium-nitrate ratio for various crops: a meta-analysis. Field Crops Res 307:109240. 10.1016/j.fcr.2023.109240

[CR7] Choi K-Y, Wernick DG, Tat CA, Liao JC (2014) Consolidated conversion of protein waste into biofuels and ammonia using *Bacillus subtilis*. Metab Eng 23:53–61. 10.1016/j.ymben.2014.02.007 24566040 10.1016/j.ymben.2014.02.007

[CR8] Colla G, Nardi S, Cardarelli M, Ertani A, Lucini L, Canaguier R, Rouphael Y (2015) Protein hydrolysates as biostimulants in horticulture. Sci Hortic 196:28–38. 10.1016/j.scienta.2015.08.037

[CR9] Costa OYA, Chang J, Li J, van Lith W, Kuramae EE (2024) Unraveling the impact of protein hydrolysates on rhizosphere microbial communities: source matters. Appl Soil Ecol 196:105307. 10.1016/j.apsoil.2024.105307

[CR10] Devi S, Kesta K, Sharma M, Chand S, Manorma K, Dilta BS, Upadhyay NK, Chauhan PK, Gupta S (2024) Effect of chicken feather hydrolysate on growth and yield of French bean. Waste Biomass Valorization 15:5387–5414. 10.1007/s12649-024-02532-1

[CR11] Duchateau S, Crouzet J, Dorey S, Aziz A (2024) The plant-associated Pantoea spp. as biocontrol agents: mechanisms and diversity of bacteria-produced metabolites as a prospective tool for plant protection. Biol Control 188:105441. 10.1016/j.biocontrol.2024.105441

[CR12] Dutkiewicz J, Mackiewicz B, Lemieszek MK, Golec M, Milanowski J (2016) Pantoea agglomerans: a mysterious bacterium of evil and good. Part IV. Beneficial effects. Ann Agric Environ Med 23(2):206–222. 10.5604/12321966.1203879 27294621 10.5604/12321966.1203879

[CR13] Gharwalova L, Paulova L, Patakova P, Branska B, Melzoch K (2018) Use of wheat straw and chicken feather hydrolysates as a complete medium for lactic acid production. Czech J Food Sci 36(2):146–153. 10.17221/461/2017-CJFS

[CR14] Girotto F, Cossu R (2017) Animal Waste: Opportunities and Challenges. In: Lichtfouse, E. (eds) Sustainable Agriculture Reviews. Sustainable Agriculture Reviews, vol 22. Springer, Cham. 2 10.1007/978-3-319-48006-0_1

[CR15] Jeong JH, Lee OM, Jeon YD, Kim JD, Lee NR, Lee CY, Son HJ (2010) Production of keratinolytic enzyme by a newly isolated feather-degrading Stenotrophomonas maltophilia that produces plant growth-promoting activity. Process Biochem 45:1738–1745. 10.1016/j.procbio.2010.07.020

[CR16] Kaur IP, Inkollu S, Prakash A, Gandhi H, Mughal MS, Du D (2020) Pantoea agglomerans bacteremia: is it dangerous? Case Reports in Infectious Diseases 7890305. 10.1155/2020/7890305 10.1155/2020/7890305 PMC7160720 32313708

[CR17] Kolek J, Driml M, Kumzak M, Soukup P, Szendzielarz F, Simera M, Branska B, Patakova P (2023) Use of common carp waste for pigment production by monascus purpureus. Kvas Prum 69(1):686–691. 10.18832/kp2023.69.686

[CR18] López-García G, Dublan-García O, Arizmendi-Cotero D, Gómez Oliván LM (2022) Antioxidant and antimicrobial peptides derived from food proteins. Molecules 27(4):1343. 10.3390/molecules27041343 35209132 10.3390/molecules27041343 PMC8878547

[CR19] López-Pedrouso M, Zaky AA, Lorenzo JM, Camiña M, Franco D (2023) A review on bioactive peptides derived from meat and by-products: extraction methods, biological activities, applications and limitations. Meat Sci 204:109278. 10.1016/j.meatsci.2023.109278 37442015 10.1016/j.meatsci.2023.109278

[CR20] Matsunaga Y, Sakata Y, Yago T, Nakamura H, Shimizu T, Takeda Y (2018) Effects of glucose with casein peptide supplementation on post-exercise muscle glycogen resynthesis in C57BL/6J mice. Nutrients 10(6):753. 10.3390/nu10060753 29891805 10.3390/nu10060753 PMC6024860

[CR21] Mishra A, Jung D, Kim NK, Bhattacharyya D (2023) Influence of chicken feather fibre processing technique on mechanical and fire performances of flame-retardant polypropylene composites. Compos - A: Appl Sci Manuf 165:107338. 10.1016/j.compositesa.2022.107338

[CR22] Nafady NA, Hassan EA, Abd-Alla MH, Bagy MMK Effectiveness of eco-friendly arbuscular mycorrhizal fungi biofertilizer and bacterial feather hydrolysate in promoting growth of Vicia faba in sandy soil. Biocata. Agric Biotechnol 16:140-7., Nakamura A, Takahashi H, Sulaiman S, Phraephaisarn C, Keeratipibul S, Kuda T, Kimura B (2018) (2021) Evaluation of peptones from chicken waste as a nitrogen source for micro-organisms. Lett Appl Microbiol 72(4):408 – 14. 10.1111/lam.13428 10.1111/lam.13428 33188703

[CR48] Nakamura A, Takahashi H, Sulaiman S, Phraephaisarn C, Keeratipibul S, Kuda T, Kimura B (2021) Evaluation of peptones from chicken waste as a nitrogen source for micro-organisms. Lett Appl Microbiol 72(4):408–14. 10.1111/lam.13428 10.1111/lam.13428 33188703

[CR23] Nirmal NP, Santivarangkna C, Rajput MS, Benjakul S, Maqsood S (2022) Valorization of fish byproducts: sources to end-product applications of bioactive protein hydrolysate. Compr Rev Food Sci Food Saf 21:1803–1842. 10.1111/1541-4337.12917 35150206 10.1111/1541-4337.12917

[CR24] Pantoea agglomerans, DBM 3797 chromosome, complete genom (2021) https://www.ncbi.nlm.nih.gov/nuccore/NZ_CP086133.1. Accessed 17 Feb 2025

[CR25] Patakova P, Vasylkivska M, Sedlar K, Jureckova K, Bezdicek M, Lovecka P, Branska B, Kastanek P, Krofta K (2024) Whole genome sequencing and characterization of *Pantoea agglomerans* DBM 3797, endophyte, isolated from fresh hop (*Humulus lupulus* L). Front Microbiol 15:1305338. 10.3389/fmicb.2024.1305338 38389535 10.3389/fmicb.2024.1305338 PMC10882544

[CR26] Paulova L, Chmelik J, Branska B, Patakova P, Drahokoupil M, Melzoch K (2020) Comparison of lactic acid production by L. casei in Batch, Fed-batch and continuous Cultivation, testing the use of feather hydrolysate as a complex nitrogen source. Braz Arch Biol Technol 63:e20190151. 10.1590/1678-4324-2020190151

[CR27] Radosavljević M, Pejin J, Pribić M, Kocić-Tanackov S, Mladenović D, Djukić-Vuković A, Mojović L (2020) Brewing and malting technology by-products as raw materials in L-(+)-lactic acid fermentation. J Chem Technol Biotechnol 95(2):339–347. 10.1002/jctb.5878

[CR28] Rajabi M, Ali A, McConnell M, Cabral J (2020) Keratinous materials: structures and functions in biomedical applications. Mater Sci Eng C 110:110612. 10.1016/j.msec.2019.110612 10.1016/j.msec.2019.110612 32204061

[CR29] Richardson AJ, McKain N, Wallace RJ (2013) Ammonia production by human faecal bacteria, and the enumeration, isolation and characterization of bacteria capable of growth on peptides and amino acids. BMC Microbiol 13:6. 10.1186/1471-2180-13-6 23312016 10.1186/1471-2180-13-6 PMC3554466

[CR30] Rouphael Y, Carillo P, Cristofano F, Cardarelli M, Colla G (2021) Effects of vegetal- versus animal-derived protein hydrolysate on sweet basil morpho-physiological and metabolic traits. Sci Hortic 284:110123. 10.1016/j.scienta.2021.110123

[CR31] Sahoo S, Rath B, Mondal KC, Halder SK, Mandal A (2023) Production optimization of feather hydrolysate and use as a promising nitogen-rich fertilizer for rice (*Oryza sativa*) production. Biosci Biotech Res Asia. 10.13005/bbra/3136

[CR32] Santi C, Zamboni A, Varanini Z, Pandolfini T (2017) Growth stimulatory effects and genome-wide transcriptional changes produced by protein hydrolysates in maize seedlings. Front Plant Sci 8:433. 10.3389/fpls.2017.00433 28424716 10.3389/fpls.2017.00433 PMC5371660

[CR33] Savijoki K, Ingmer H, Varmanen P (2006) Proteolytic systems of lactic acid bacteria. Appl Microbiol Biotechnol 71(4):394–406. 10.1007/s00253-006-0427-1 16628446 10.1007/s00253-006-0427-1

[CR34] Sezonov G, Joseleau-Petit D, D’Ari R (2007) *Escherichia coli* physiology in Luria-Bertani broth. J Bacteriol 189(23):8746–8749. 10.1128/jb.01368-07 17905994 10.1128/JB.01368-07 PMC2168924

[CR35] Smirnova TA, Viskin A, Hoskova M, Habartova L, Setnicka V, Cejnar P, Kuckova S (2021) Comparison of proteomic approaches used for the detection of potential biomarkers of Alzheimer’s disease in blood plasma. J Sep Sci 44(22):4132–4140. 10.1002/jssc.202100468 34545700 10.1002/jssc.202100468

[CR36] Solcova O, Knapek J, Wimmerova L, Vavrova K, Kralik T, Rouskova M, Sabata S, Hanika J (2021) Environmental aspects and economic evaluation of new green hydrolysis method for waste feather processing. Clean Techn Environ Policy 23(6):1863–1872. 10.1007/s10098-021-02072-5

[CR37] Stiborova H, Branska B, Vesela T, Lovecka P, Stranska M, Hajslova J, Jiru M, Patakova P, Demnerova K (2016) Transformation of raw feather waste into digestible peptides and amino acids. J Chem Technol Biotechnol 91(6):1629–1637. 10.1002/jctb.4912

[CR38] Talha M, Tanveer M, Abid A, Maan AA, Khan MKI, Shair H, Tanveer N, Mustafa A (2024) Valorization of poultry slaughter wastes via extraction of three structural proteins (gelatin, collagen and keratin): a sustainable approach for circular economy. Trends Food Sci Technol 152:104667. 10.1016/j.tifs.2024.104667

[CR39] Tatemichi Y, Kuroda K, Nakahara T, Ueda M (2020) Efficient ammonia production from food by-products by engineered *Escherichia coli*. AMB Expr 10(1):150. 10.1186/s13568-020-01083-7 10.1186/s13568-020-01083-7 PMC7434829 32809073

[CR40] Tomé D (2021) Yeast extracts: nutritional and flavoring food ingredients. ACS Food Sci Technol 1(4):487–494. 10.1021/acsfoodscitech.0c00131

[CR41] Vavrova K, Wimmerova L, Knapek J, Weger J, Keken Z, Kastanek F, Solcova O (2022) Waste feathers processing to liquid fertilizers for sustainable agriculture—LCA, economic evaluation, and case study. Processes 10(12):2478. 10.3390/pr10122478

[CR42] Vélez-Erazo EM, Saturno RP, Marson GV, Hubinger MD (2021) Spent brewer’s yeast proteins and cell debris as innovative emulsifiers and carrier materials for edible oil microencapsulation. Food Res Int 140:109853. 10.1016/j.foodres.2020.109853 33648171 10.1016/j.foodres.2020.109853

[CR43] Ward BK, Dufault RJ, Hassell R, Cutulle MA (2018) Affinity of hyperammonia-producing bacteria to produce bioammonium/ammonia utilizing five organic nitrogen substrates for potential use as an organic liquid fertilizer. ACS Omega 3(9):11817–11822. 10.1021/acsomega.7b02083 30320275 10.1021/acsomega.7b02083 PMC6173566

[CR44] Watanabe Y, Kuroda K, Tatemichi Y, Nakahara T, Aoki W, Ueda M (2020) Construction of engineered yeast producing ammonia from glutamine and soybean residues (okara). AMB Expr 10(1):70. 10.1186/s13568-020-01011-9 10.1186/s13568-020-01011-9 PMC7158961 32296960

[CR45] Xiang Y, Peng J, Shao Y, Son JE, Tagawa K, Yamada S, Yamada M, Baiyin B, Yang Q (2025) Auxin responds to flowing nutrient solution to accelerate the root growth of lettuce in hydroponic culture. Int J Mol Sci 26(16):7742. 10.3390/ijms26167742 40869068 10.3390/ijms26167742 PMC12386315

[CR46] Zhang YM, Ye DX, Liu Y, Zhang XY, Zhou YL, Zhang L, Yang XL (2023) Peptides, new tools for plant protection in eco-agriculture. Adv Agrochem 2:58–78. 10.1016/j.aac.2023.01.003

[CR47] Zhao CC, Eun JB (2018) Isolation and identification of hyper-ammonia-producing bacteria from commercial fermented skates (*Raja kenojei*). J Food Sci Technol 55(12):5082–5090. 10.1007/s13197-018-3447-9 30483004 10.1007/s13197-018-3447-9 PMC6233455

