# Supplementary material for: Ammonia release from hydrolysed animal and food waste by the hop endophyte, Pantoea agglomerans
Source: World J Microbiol Biotechnol. 2025 Dec 2;41(12):494. doi: 10.1007/s11274-025-04720-0 (PMC12672727; doi:10.1007/s11274-025-04720-0)
Supplement: Supplementary file 1 — Supplementary Material 1 (DOCX 7.09 MB) [file 11274_2025_4720_MOESM1_ESM.docx]

**Ammonia Release from Hydrolysed Animal and Food Waste by the Hop Endophyte, *Pantoea agglomerans***

**Tomas Hasek^a^, Frantisek Kastanek^b^, Tatiana Anatolievna Smirnova^c^, Olga Solcova^b^, Stepanka Kuckova^c^, Barbora Branska^a^, Karel Melzoch^a^, Petra Patakova^a*^**

^a^Department of Biotechnology, University of Chemistry and Technology Prague, Technicka 5, 166 28 Prague 6 Czechia
^b^Research Group of Catalysis and Reaction Engineering, Institute of Chemical Process Fundamentals of the Czech Academy of Sciences, Rozvojova 135, 165 00, Prague 6, Czechia
^c^Department of Biochemistry and Microbiology, University of Chemistry and Technology Prague, Technicka 3, 166 28 Prague 6, Czechia

***** Corresponding author: Petra Patakova, Department of Biotechnology, University of Chemistry and Technology Prague, Technicka 5, 166 28 Prague 6, Czechia. Email: [petra.patakova@vscht.cz](mailto:petra.patakova@vscht.cz)

**Supplement Table 1a Analysis of Statistical Significance of Bacterial Specific Growth Rate Results** The table shows the results of the comparison of the counted bacterial specific growth rate of each possible pair of hydrolysates according to the Duncan correction method. Half of them are statistically different, and in the second half of them, as shown below, the difference between the growth rates was not significant.

|  | N | a | b | c | d | e | f |
| --- | --- | --- | --- | --- | --- | --- | --- |
| Feather acid hydrolysate (D) | 5 | 0.11 |  |  |  |  |  |
| Feather alkaline hydrolysate (F) | 5 |  | 0.19 |  |  |  |  |
| Chicken leftover acid hydrolysate (C) | 5 |  | 0.22 | 0.22 |  |  |  |
| Spent brewer’s yeast autolyzate (G) | 5 |  |  | 0.25 | 0.25 |  |  |
| LB medium | 5 |  |  | 0.27 | 0.27 | 0.27 |  |
| Chicken leftover enzyme hydrolysate (B) | 3 |  |  |  | 0.28 | 0.28 |  |
| Chicken leftover acid hydrolysate (A) | 4 |  |  |  |  | 0.31 |  |
| Carp residues acid hydrolysate (E) | 5 |  |  |  |  |  | 0.63 |
| Sig. |  | 1.000 | 0.292 | 0.065 | 0.300 | 0.146 | 1.000 |
| Means for groups in homogeneous subsets are displayed. Based on observed means.  The error term is Mean Square(Error) = 0.001. a. Uses Harmonic Mean Sample Size = 4.486. b. The group sizes are unequal. The harmonic mean of the group sizes is used. Type I error levels are not guaranteed. c. Alpha = 0.05. | | | | | | | |

**Supplement Table 1b Analysis of Statistical Significance of Bacterial Lag Phase Results** The table shows the results of the comparison of the counted bacterial lag phase of each possible pair of hydrolysates according to the Duncan correction method.

|  | N | a | b | c | d |
| --- | --- | --- | --- | --- | --- |
| Feather alkaline hydrolysate (F) | 5 | 3.30 |  |  |  |
| LB medium | 5 | 3.60 |  |  |  |
| Feather acid hydrolysate (D) | 5 |  | 5.10 |  |  |
| Chicken leftover enzyme hydrolysate (B) | 3 |  | 5.33 |  |  |
| Carp residues acid hydrolysate (E) | 5 |  | 5.50 | 5.50 |  |
| Spent brewer’s yeast autolyzate (G) | 5 |  |  | 5.80 |  |
| Chicken leftover acid hydrolysate (A) | 4 |  |  |  | 6.25 |
| Chicken leftover acid hydrolysate (C) | 5 |  |  |  | 6.50 |
| Sig. |  | 0.130 | 0.058 | 0.130 | 0.205 |
| Means for groups in homogeneous subsets are displayed. Based on observed means. The error term is Mean Square(Error) = 0.083. a. Uses Harmonic Mean Sample Size = 4.486. b. The group sizes are unequal. The harmonic mean of the group sizes is used. Type I error levels are not guaranteed. c. Alpha = 0.05. | | | | | |

**Supplement Table 1c Analysis of Statistical Significance of Bacterial Log Phase Results** The table shows the results of the comparison of the counted bacterial log phase of each possible pair of hydrolysates according to the Duncan correction method.

|  | N | a | b | c | d |
| --- | --- | --- | --- | --- | --- |
| Feather acid hydrolysate (D) | 5 | 5.20 |  |  |  |
| Chicken leftover enzyme hydrolysate (B) | 3 | 5.50 |  |  |  |
| Carp residues acid hydrolysate (E) | 5 | 6.10 |  |  |  |
| Feather alkaline hydrolysate (F) | 5 |  | 7.90 |  |  |
| Spent brewer’s yeast autolyzate (G) | 5 |  | 8.20 | 8.20 |  |
| Chicken leftover acid hydrolysate (A) | 4 |  | 8.88 | 8.88 |  |
| LB medium | 5 |  |  | 9.20 |  |
| Chicken leftover acid hydrolysate (C) | 5 |  |  |  | 11.20 |
| Sig. |  | 0.079 | 0.057 | 0.052 | 1.000 |
| Means for groups in homogeneous subsets are displayed. Based on observed means. The error term is Mean Square(Error) = 0.493. a. Uses Harmonic Mean Sample Size = 4.486. b. The group sizes are unequal. The harmonic mean of the group sizes is used. Type I error levels are not guaranteed. c. Alpha = 0.05. | | | | | |

**Supplement Table 1d Analysis of Statistical Significance of Optical Density Results** The table shows the results of the comparison of the optical density measured after 22 hours from inoculation of each possible pair of hydrolysates according to the Duncan correction method. Almost all of them are statistically different, but in some cases, as shown below, the difference between the concentrations was not significant (hydrolysates D with G).

|  | N | a | b | c | d | e | f | g |
| --- | --- | --- | --- | --- | --- | --- | --- | --- |
| Chicken leftover acid hydrolysate (A) | 4 | 0.45 |  |  |  |  |  |  |
| Feather alkaline hydrolysate (F) | 5 |  | 0.56 |  |  |  |  |  |
| Carp residues acid hydrolysate (E) | 5 |  |  | 0.74 |  |  |  |  |
| Chicken leftover enzyme hydrolysate (B) | 3 |  |  |  | 0.89 |  |  |  |
| Chicken leftover acid hydrolysate (C) | 5 |  |  |  |  | 0.99 |  |  |
| Spent brewer’s yeast autolyzate (G) | 5 |  |  |  |  |  | 1.25 |  |
| Feather acid hydrolysate (D) | 5 |  |  |  |  |  | 1.28 |  |
| LB medium | 5 |  |  |  |  |  |  | 1.36 |
| Sig. |  | 1.000 | 1.000 | 1.000 | 1.000 | 1.000 | 0.052 | 1.000 |
| Means for groups in homogeneous subsets are displayed. Based on observed means. The error term is Mean Square(Error) = 0.000.  a. Uses Harmonic Mean Sample Size = 4.486. b. The group sizes are unequal. The harmonic mean of the group sizes is used. Type I error levels are not guaranteed. c. Alpha = 0.05. | | | | | | | | |
|  | | | | | | | | |

**Supplement Table 2a Analysis of Statistical Significance of Ammonia Release Results** The table shows the results of the comparison of the measured ammonium ion concentrations of each possible pair of hydrolysates according to the Duncan correction method. Almost all of them are statistically different, but in some cases, as shown below, the difference between the concentrations was not significant (hydrolysates F and G).

|  | N | a | b | c | d | e | f | g |
| --- | --- | --- | --- | --- | --- | --- | --- | --- |
| LB medium | 3 | 215.88 |  |  |  |  |  |  |
| Chicken leftover acid hydrolysate (A) | 3 |  | 262.78 |  |  |  |  |  |
| Carp residues acid hydrolysate (E) | 3 |  |  | 502.11 |  |  |  |  |
| Chicken leftover acid hydrolysate (C) | 3 |  |  |  | 707.17 |  |  |  |
| Chicken leftover enzyme hydrolysate (B) | 3 |  |  |  |  | 773.92 |  |  |
| Feather alkaline hydrolysate (F) | 3 |  |  |  |  |  | 824.24 |  |
| Spent brewer’s yeast autolyzate (G) | 3 |  |  |  |  |  | 828.64 |  |
| Feather acid hydrolysate (D) | 3 |  |  |  |  |  |  | 943.49 |
| Sig. |  | 1.000 | 1.000 | 1.000 | 1.000 | 1.000 | 0.842 | 1.000 |
| Means for groups in homogeneous subsets are displayed. Based on observed means. The error term is Mean Square(Error) = 709.076. a. Uses Harmonic Mean Sample Size = 3.000. b. Alpha = 0.05. | | | | | | | | |

**Supplement Table 2b Analysis of Statistical Significance of pH Values** The table shows the results of the comparison of the measured pH values of each possible pair of hydrolysates according to the Duncan correction method. Most of them are statistically different, but in some cases, as shown below, the difference between the pH values was not significant (hydrolysates D with E, the hydrolysate E with G and LB medium).

|  | N | a | b | c | d | e | f |
| --- | --- | --- | --- | --- | --- | --- | --- |
| Chicken leftover enzyme hydrolysate (B) | 3 | 7.95 |  |  |  |  |  |
| Chicken leftover acid hydrolysate (A) | 3 |  | 8.37 |  |  |  |  |
| Chicken leftover acid hydrolysate (C) | 3 |  |  | 8.45 |  |  |  |
| Feather acid hydrolysate (D) | 3 |  |  |  | 8.56 |  |  |
| Carp residues acid hydrolysate (E) | 3 |  |  |  | 8.59 | 8.59 |  |
| Spent brewer’s yeast autolyzate (G) | 3 |  |  |  |  | 8.63 |  |
| LB medium | 3 |  |  |  |  | 8.64 |  |
| Feather alkaline hydrolysate (F) | 3 |  |  |  |  |  | 9.03 |
| Chicken leftover enzyme hydrolysate (B) |  | 1.000 | 1.000 | 1.000 | 0.144 | 0.081 | 1.000 |
| Means for groups in homogeneous subsets are displayed. Based on observed means. The error term is Mean Square(Error) = 0.001. a. Uses Harmonic Mean Sample Size = 3.000. b. Alpha = 0.05. | | | | | | | |

**Supplement Table 2c Analysis of Statistical Significance of Ammoinum Ions Concentration per Gram of Feedstock** The table shows the results of the comparison of the calculated ammonium ions concentration per gram of feedstock of each possible pair of hydrolysates according to the Duncan correction method. Most of them are statistically different, but in some cases, as shown below, the difference between the concentrations was not significant (hydrolysates G with A, the hydrolysate D with B).

|  | N | a | b | c | d | e | f |
| --- | --- | --- | --- | --- | --- | --- | --- |
| Spent brewer’s yeast autolyzate (G) | 3 | 1.66 |  |  |  |  |  |
| Chicken leftover acid hydrolysate (A) | 3 | 1.88 |  |  |  |  |  |
| Carp residues acid hydrolysate (E) | 3 |  | 3.77 |  |  |  |  |
| Chicken leftover acid hydrolysate (C) | 3 |  |  | 5.30 |  |  |  |
| Feather acid hydrolysate (D) | 3 |  |  |  | 7.08 |  |  |
| Chicken leftover enzyme hydrolysate (B) | 3 |  |  |  | 7.74 |  |  |
| LB medium | 3 |  |  |  |  | 14.39 |  |
| Feather alkaline hydrolysate (F) | 3 |  |  |  |  |  | 41.40 |
| Sig. |  | 0.701 | 1.000 | 1.000 | 0.256 | 1.000 | 1.000 |
| Means for groups in homogeneous subsets are displayed. Based on observed means. The error term is Mean Square(Error) = 0.475. a. Uses Harmonic Mean Sample Size = 3.000. b. Alpha = 0.05. | | | | | | | |

**Supplement Table 3 Analysis of Statistical Significance of Lettuce Growth Experiments**

Statistical analysis was performed for each part of the measurements with Duncan correction.

| Fresh Lettuce Mass | N | a | b |
| --- | --- | --- | --- |
| Water | 6 | 7.11 |  |
| Feather alkaline hydrolysate (F) | 6 |  | 8.39 |
| Murashige Skoog | 6 |  | 9.01 |
| Sig. |  | 1.000 | 0.278 |
| Means for groups in homogeneous subsets are displayed. Based on observed means. The error term is Mean Square(Error) = 0.910. a. Uses Harmonic Mean Sample Size = 6.000. b. Alpha = 0.05. | | | |

| Dry Lettuce Mass | N | a |
| --- | --- | --- |
| Water | 6 | 0.34 |
| Feather alkaline hydrolysate (F) | 6 | 0.37 |
| Murashige Skoog | 6 | 0.40 |
| Sig. |  | 0.188 |
| Means for groups in homogeneous subsets are displayed. Based on observed means. The error term is Mean Square(Error) = 0.005. a. Uses Harmonic Mean Sample Size = 6.000. b. Alpha = 0.05. | | |

| Number of Leaves | N | a | b | c |
| --- | --- | --- | --- | --- |
| Water | 5 | 13.00 |  |  |
| Feather alkaline hydrolysate (F) | 5 |  | 14.50 |  |
| Murashige Skoog | 5 |  |  | 15.33 |
| Sig. |  | 1.000 | 1.000 | 1.000 |
| Means for groups in homogeneous subsets are displayed. Based on observed means. The error term is Mean Square(Error) = 0.456. a. Uses Harmonic Mean Sample Size = 6.000. b. Alpha = 0.05. | | | | |

| Root Length | N | a | b |
| --- | --- | --- | --- |
| Water | 6 | 8.17 |  |
| Feather alkaline hydrolysate (F) | 6 | 8.47 |  |
| Murashige Skoog | 6 |  | 12.33 |
| Sig. |  | 0.829 | 1.000 |
| Means for groups in homogeneous subsets are displayed. Based on observed means. The error term is Mean Square(Error) = 5.623. a. Uses Harmonic Mean Sample Size = 6.000. b. Alpha = 0.05. | | | |

**
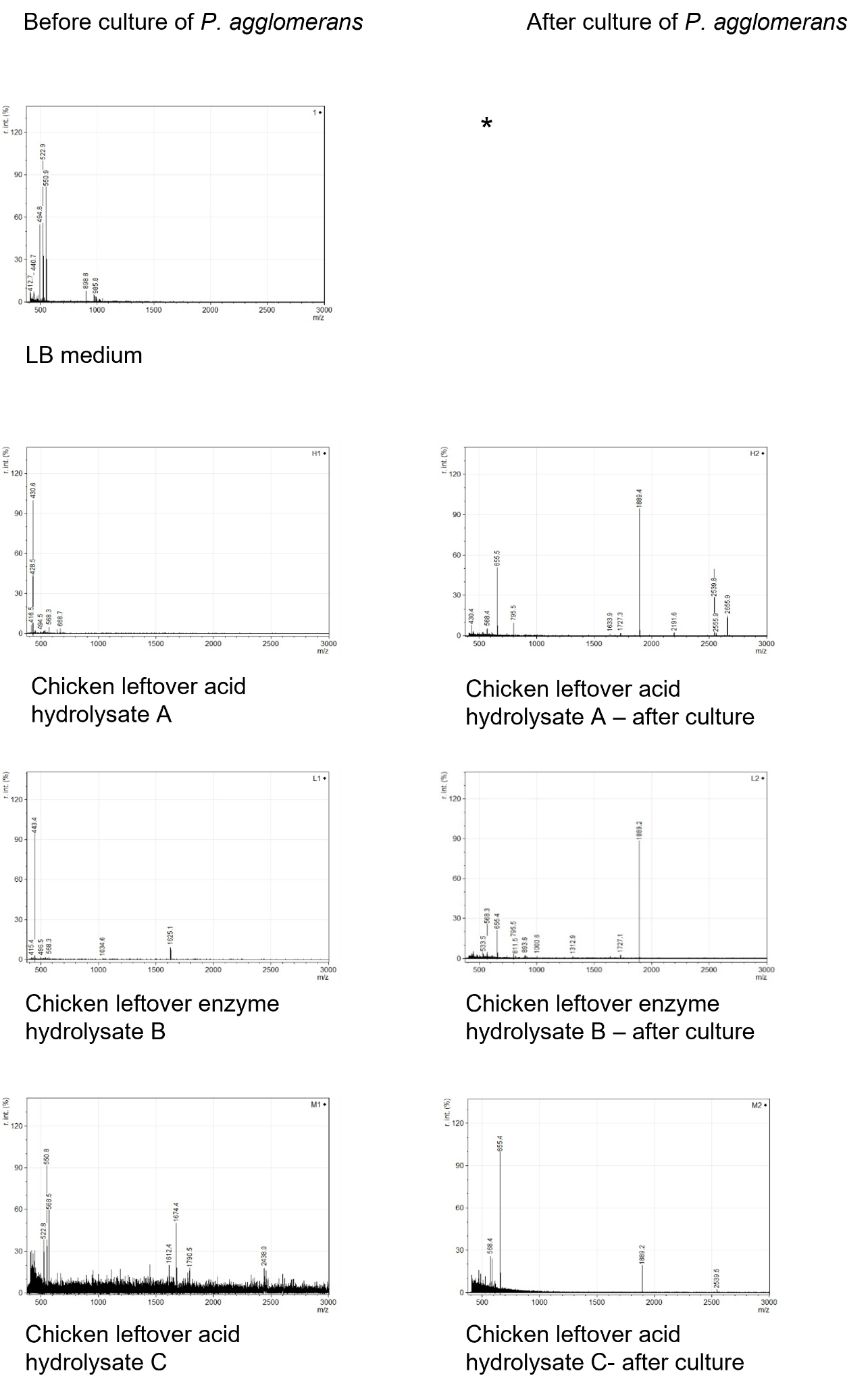
**

**
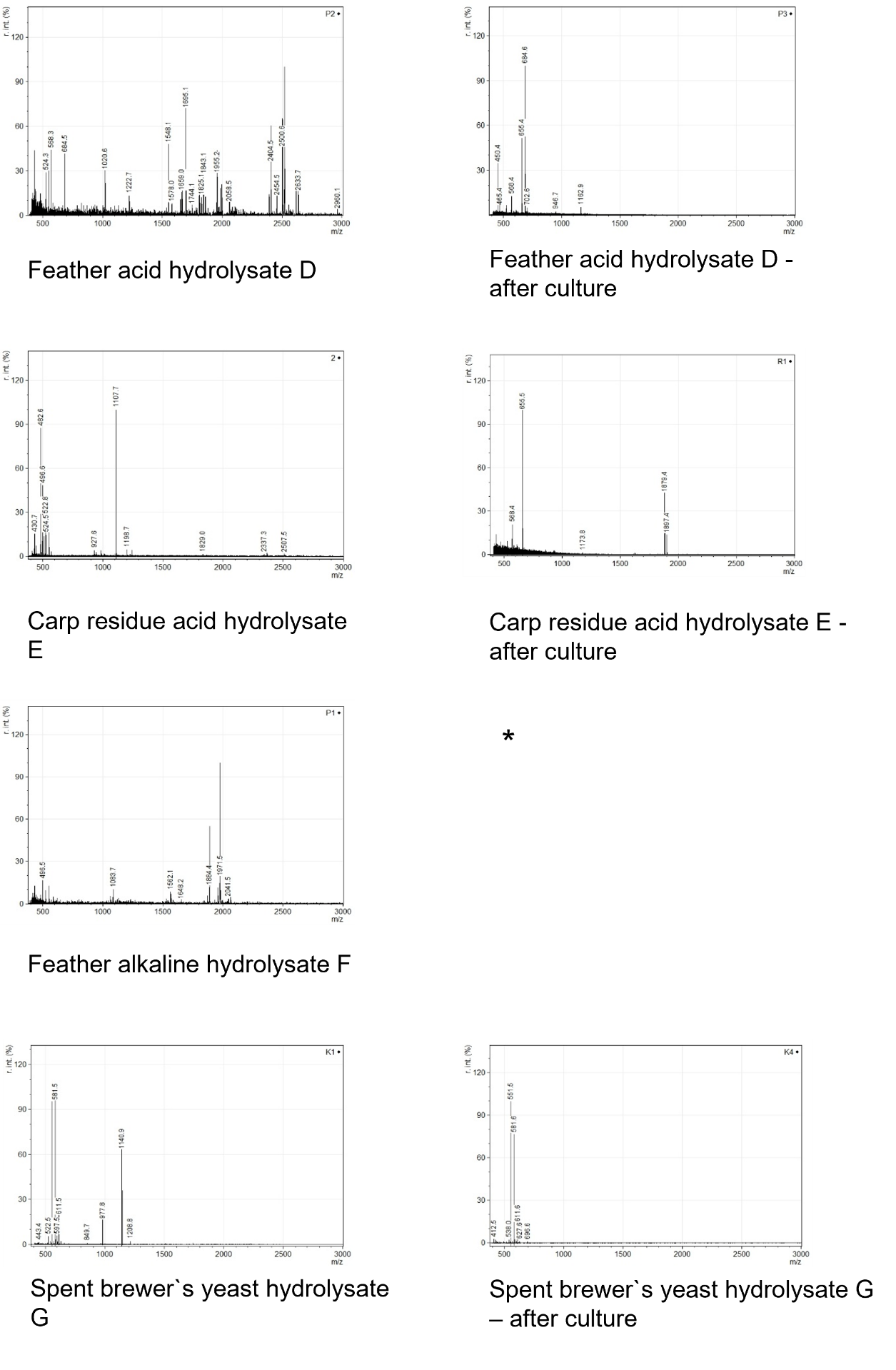
**

**Supplement Figure 1 MALDI-TOF mass spectra showing the hydrolysate (left picture in a row) and hydrolysate after cultivation with *Pantoea agglomerans* DBM 3797 (right picture).**

*In case of LB medium and alkaline feather hydrolysate (F) after cultivation, the spectra are not available.


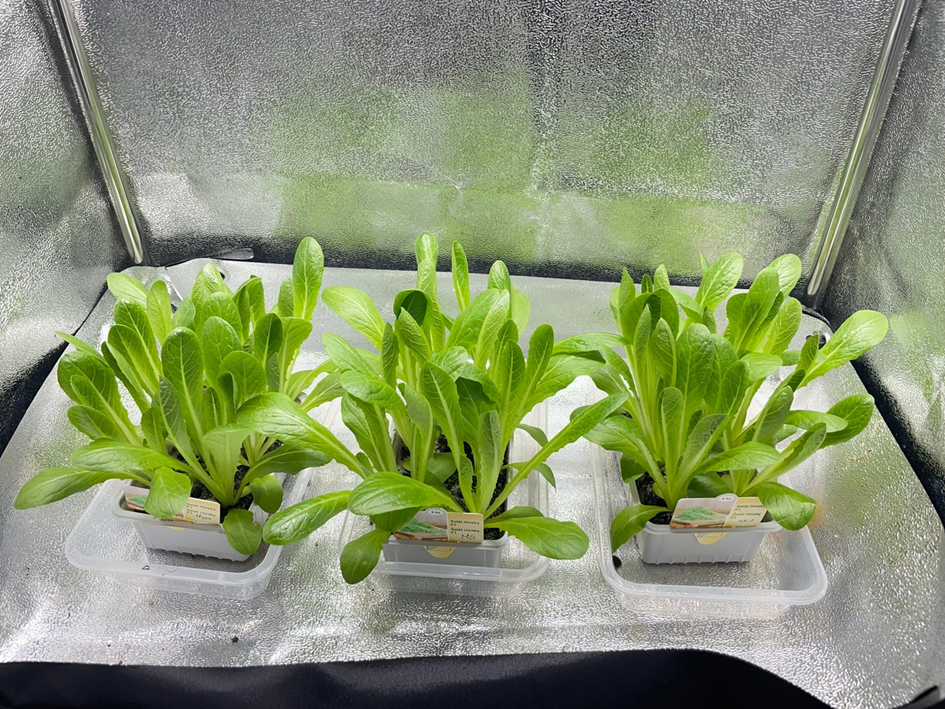


**A)**

**B)**

**C)**

**Supplement Figure 2:** **Performance of the growth experiment**

Three groups of lettuce (*Lactuca sativa* var. *Longifolia)* seedlings, each having 6 individual plants that were grown under standard conditions (simulated 12 h day, temperature 26-28 °C, humidity of 70-80 %) for one week in the greenhouse tent. In each group of seedlings, individual plants were watered by syringe into the soil with 10 mL of the different agent: A) distilled water, B) Murashige Skoog Basal Salt Mixture (control), C) the diluted feather hydrolysate with *P. agglomerans*.


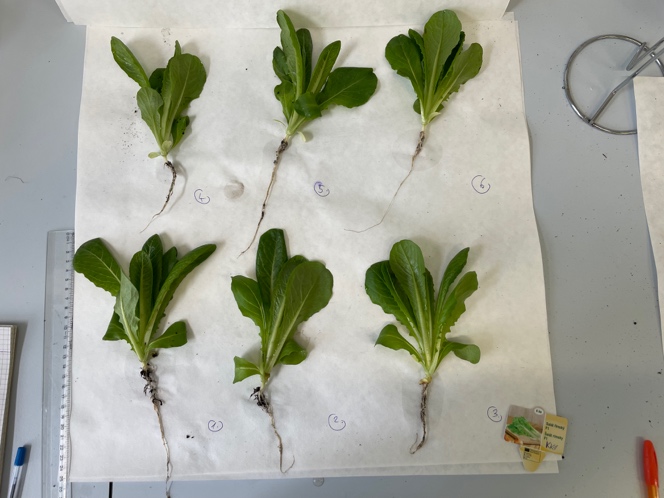

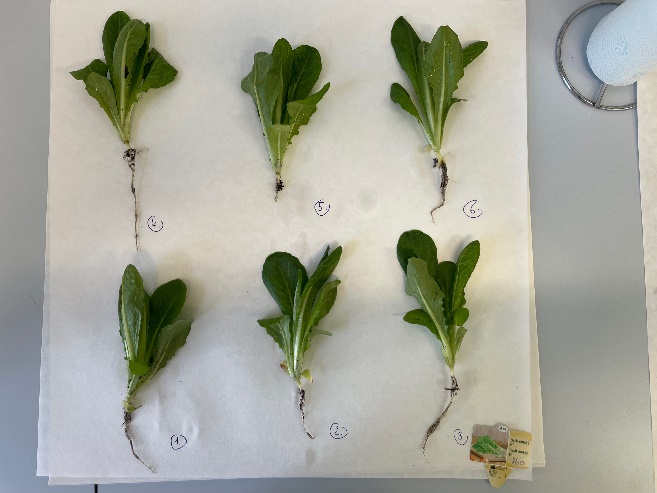

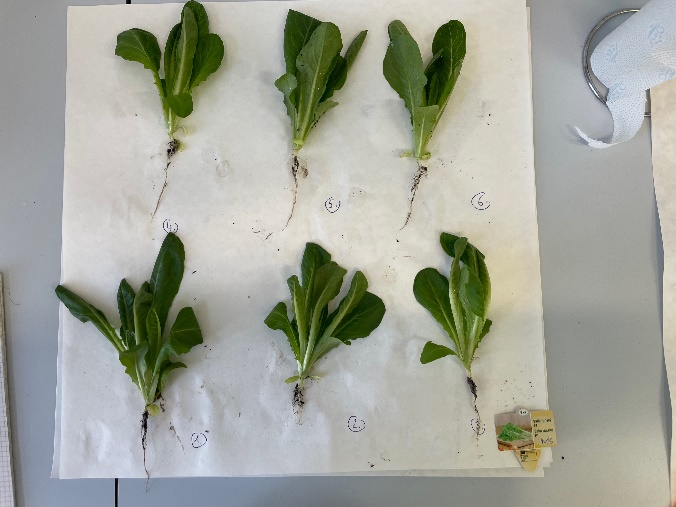


**A)**

**B)**

**C)**

**Supplement Figure 3:** **The root length comparison**

The root lengths of three groups of lettuce were compared. In each group of seedlings, individual plants were watered by syringe into the soil with 10 mL of the different agent: A) distilled water, B) Murashige Skoog Basal Salt Mixture (control), C) the diluted feather hydrolysate with *P. agglomerans*.
